# Wearable-derived skin temperature dynamics during sleep reveal cardiovascular perfusion deficits through mechanistic modeling

**DOI:** 10.1038/s41746-026-02633-2

**Published:** 2026-04-22

**Authors:** Jamison H. Burks, Wendy Hartogensis, Stephan Dilchert, Ashley E. Mason, Benjamin L. Smarr

**Affiliations:** 1 Shiu Chen – Gene Lay Department of Bioengineering, Jacobs School of Engineering, University of California, San Diego, La Jolla, CA USA; 2 Osher Center for Integrative Health, University of California, San Francisco, CA USA; 3 Department of Management, Zicklin School of Business, Baruch College, The City University of New York, New York, NY USA; 4 Halicioğlu Data Science Institute, University of California, San Diego, La Jolla, CA USA

**Keywords:** Computational biology and bioinformatics, Mathematics and computing, Physiology

## Abstract

Classical statistics are commonly used to find differences between distributions of average skin temperature across populations. However, skin temperature is affected by many endogenous (within body) and exogenous (outside body) factors, and these factors induce causal changes in longitudinal skin temperature that can obfuscate the interpretation of average population differences. Moreover, interpretations are increasingly difficult to make when using temperature signals sampled longitudinally in uncontrolled settings. A potential way to better handle the inherent complexity of skin temperature dynamics in uncontrolled settings is to explicitly account for the effects of causal factors on the short- and long-term trajectories of temperature. In this work, we find that a physics-informed model of skin temperature and activity during sleep accounts for significantly more variance than an equally parsimonious linear model. Furthermore, this model enables separation of cohorts with cardiovascular conditions that are known to affect skin thermoregulation, an important improvement over classic statistical modeling.

## Introduction

Skin temperature is influenced by various factors, including thyroid and endocrine disorders^1,2^, infections^3,4^, mental health^5,6^, and aging^7^. This suggests that, with appropriate signal processing, continuous skin temperature measurement could reveal signs of physiological changes associated with factors relevant to overall health. If altered temperature patterns related to such factors are discernable from skin temperature time series data, then it may be possible to use skin temperature as an easily accessible biomarker to support screening, facilitating identification of conditions that affect thermoregulatory control in outpatient settings.

The proliferation of wearable devices presents an opportunity to monitor skin temperature passively across large, diverse populations under real-world conditions^8–10^. This capability holds promise for early detection of various health conditions^11–15^. However, the uncontrolled environment in which wearable data are generated introduces significant challenges to analyzing skin temperature measurements. Specifically, skin temperature varies over time due to a combination of influences such as hormonal fluctuations^16,17^, core body temperature^1,18,19^, ambient temperature^18,20,21^, adiposity^22,23^, sweat^24–26^, movement^6^, and vasodilation^27^. These influences make it difficult to separate changes in skin temperature linked to chronic conditions from those driven by acute external factors, principal being physical activity. Another major challenge is that sequential skin temperature measurements are not independent. Readings are influenced by prior states and ongoing conditions, making them interdependent^28^. This interdependence complicates the interpretation of temperature trends over time, especially when attempting to associate them with specific health conditions.

A potential solution to these challenges is to shift from treating skin temperature data as independent measurements over time to viewing skin temperature as part of a dynamic system, which can best be understood in the broader context of multiple factors that play a role in temperature regulation. Doing so allows us to analyze the interplay of biological and environmental influences on temperature changes^29^. This dynamic approach leverages time-series analysis, enabling us to model how temperature evolves over time and to separate short-term fluctuations (e.g., acute effects such as vasodilation/vasoconstriction, changes in ambient temperature, etc.) from meaningful long-term dynamics (e.g., circadian/ultradian rhythms, as well as the effect of extended physical activity on skin temperature changes).

In this paper, we attempt a first evaluation of this approach in uncontrolled, human skin temperature data sampled from a wearable device. To do so, we use tools designed to analyze sequential data dynamics without assuming independence between measurements. These tools enable us to model how each temperature reading relates to prior values while accounting for influencing factors. Specifically, we analyze distal skin temperature (*T*) and metabolic equivalents (MET) measured every minute during sleep — a low-activity state with intermittent movement^30^ — to assess our ability to detect temperature perturbations resulting from MET as a single model cause. MET, commonly used to estimate metabolic activity^31^, is approximated here using movement data captured by wearable accelerometers.

While true MET reflects metabolic activity and should remain low during sleep, wearable devices often capture movement-induced variability, which affects skin temperature. If our framework is correct, then by integrating these MET estimates into our model we could “de-noise” temperature data - that is, separate noise caused by external perturbations from biologically relevant changes associated with endogenous thermoregulatory mechanisms. This approach uses tools such as autoregressive modeling^32–34^ and causal inference^35,36^ techniques to quantify these relationships and isolate meaningful digital biomarkers associated with peripheral temperature regulation.

This model offers two key benefits: (1) Filtered temperature data**:** By accounting for the influence of MET through regression-based adjustment, we can isolate residual temperature changes that may be linked directly to activity perturbations, distinguishing them from temperature changes due to other causes. (2) Model parameters: We define model parameters that represent underlying physical processes, helping us identify individual differences in temperature dynamics. For example, one parameter might describe how quickly a person’s temperature recovers after a movement-induced spike in MET, and this parameter might then have implications for their cardiovascular resiliency.

**Fig. 1:**
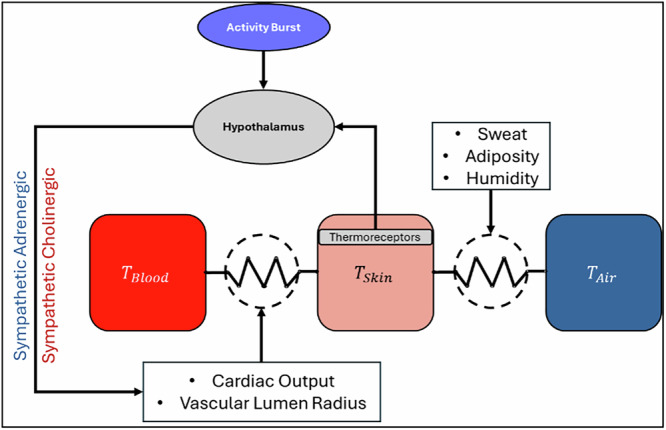
Simplified compartment model of skin thermoregulatory control. Compartments for blood (red), skin (orange), and air (blue) are physically connected to transfer thermal energy. The jagged lines between compartments are the “resistors” that affect the rate at which thermal energy can be transferred. Factors that affect these rates are shown in lists that point to them. In skin, thermoreceptors activate the hypothalamus via afferent pathways that can in turn elicit efferent vasoconstriction (sympathetic adrenergic) or vasodilation (sympathetic cholinergic). The activity burst is proposed to elicit a downstream vasoconstrictive effect.

Skin temperature is a highly dynamic variable that is dependent on core body temperature, blood temperature, and ambient temperature (Fig. 1). Masses with these temperatures act as heat stores and sinks that transfer thermal energy between each other in a state-dependent manner—the larger the difference between two heat stores, the greater the magnitude of their heat transfer rate. This heat transfer rate is also dependent on the physiological and environmental factors that can promote or inhibit the ability for any two masses to transfer thermal energy in the first place. Between the skin and air, these factors include sweat^24,25^, adiposity^22^, and humidity^37^. Between the blood and skin, these factors are largely driven by cardiac output^38^ and the local vascular lumen radius^39,40^. A greater cardiac output means that warm blood is able to replace cooler blood at a greater rate, but the access that warmer blood has to the skin can be substantially limited if epidermal microvasculature undergoes vasoconstriction—essentially causing the blood to have a propensity to move through the larger vasculature that is further from the epidermis. Vasoconstriction would therefore have an effect of lowering skin temperature by reducing blood flow to the epidermis, since there is still heat being transferred from the skin to the air (assuming the ambient temperature and the thermal conductance between the skin and air have not changed). Vascular tone is mediated by hormones^41^, neurotransmitters^42^, and locally produced chemicals^43^. Norepinephrine release, controlled by the sympathetic adrenergic pathway, is capable of inducing an acute, transient effect of vasoconstriction on the baseline vascular tone of an individual^44–47^. We proposed that transient movements, as measured by the finger-worn Oura Ring, elicit a sympathetic input that ultimately triggers downstream sympathetic adrenergic vasoconstriction that manifests as the deflection and subsequent recovery dynamics of skin temperature presented in this work. The dynamics of such a vasoconstrictive activation would likely be affected by pathologies that affect (1) the signal transduction of an activity burst into a vasoconstrictive response and (2) arterial pliability and recovery. We chose atrial fibrillation, hypertension, diabetes, and coronary artery disease (CAD) as conditions that may affect the value of the dynamical biomarkers for the temperature recovery and AP trajectories due to their associations with peripheral blood perfusion deficiencies^21,27,39,48^.

Our findings indicate that incorporating MET into a causal model accounts for the external influences of activity bursts on temperature during sleep. This enables us to focus on residual temperature changes and model parameters, which can help differentiate individuals with and without conditions that affect thermoregulation^39,48–51^. Additionally, these parameters allow for a more nuanced classification of individuals, not just based on absolute temperature values but on how their temperature dynamics respond to perturbations over time. While other modalities, such as photoplethysmography (PPG) or electrocardiography (ECG), have been used to identify cardiovascular conditions from wearables, they are often very prone to movement and other artefacts, making their signals noisy in uncontrolled settings. In this work, we show that temperature dynamics can be used as an alternative, additional signal to capture covariates of cardiovascular risk separately from other modalities. This approach demonstrates the potential of using skin temperature as a practical biomarker for cardiovascular health monitoring and disease detection, laying the groundwork for broader clinical applications in at-risk populations.

## Results

### Pearson’s correlations of activity and temperature during sleep do not capture their directional relationship

**Fig. 2:**
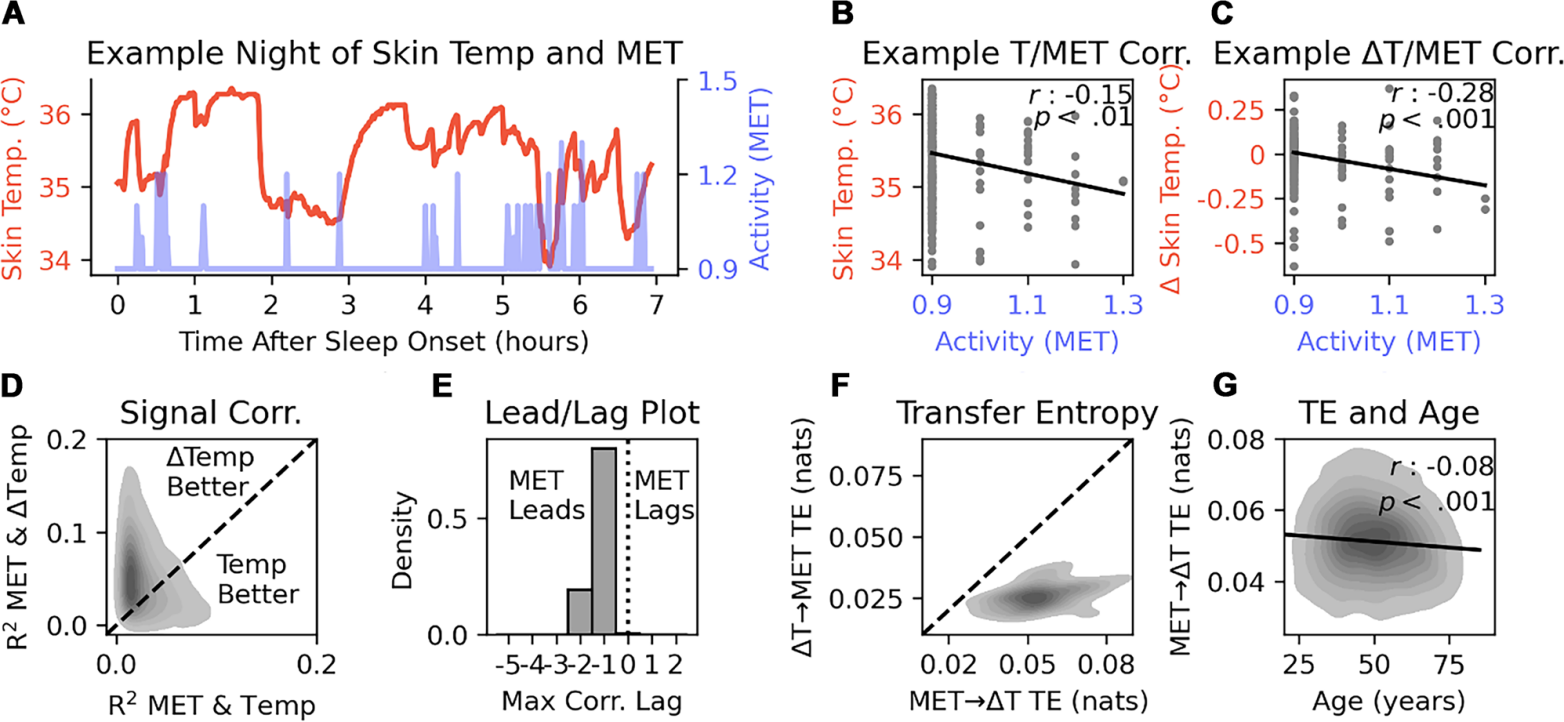
Example night of data and its associated correlations and likely time lag relationship. **A** Example night of data for *T* (red) and MET (blue) from one participant. **B** Same data as in A; example Pearson’s correlation between *T* and MET. **C** Same data as in **A**; example Pearson’s correlation between the minute-to-minute change in temperature (Δ*T*) and MET. **D** Kernel density estimate (KDE) of median *R*^2^ for each participant’s nights between using either *T* or Δ*T* and MET. Dashed identity line represents no difference; above line indicates that Δ*T* is a better covariate than *T*. **E** Lead/Lag plot of MET and Δ*T* based on the index of MET relative to Δ*T* with the greatest absolute correlation. Negative values indicate MET leads Δ*T*. **F** KDE of median TE for each participant’s nights between MET to Δ*T* (*x*-axis) and Δ*T* to MET (*y*-axis). Dashed identity line represents no difference; below line indicates MET provides more information about future Δ*T* than Δ*T* about future MET. **G** KDE of the MET to Δ*T* transfer entropy against age. Solid line is the linear fit to the data.

The initial relationship to consider is that of a linear correlation between MET and *T*. A single example of MET and *T* during a period of sleep for one participant is shown in Fig. 2A. There are two key assumptions that are often made when performing a Pearson’s correlation: (1) data on both variables are normally distributed and (2) the data are not autocorrelated (each sample in time is independent of other values in time). Visually, we observed different shapes in the trajectories of these data. The median and mode for MET were both its *minimum* value of 0.9 (Fig. 2A, Blue), a strong indication of non-normality. There were also times in which there were activity bursts in sequence – non-minimum MET values often clustered together in time (a sign of autocorrelation). The temperature data’s median was not equal to either the maximum or minimum values, making it more similar to a normal distribution; however, the data showed signs of strong autocorrelation: the next observed temperature was strongly dependent on the prior observed temperature.

Even though these data did not meet the prerequisites for performing Pearson’s correlation, it was still possible to identify significant relationships with enough data (*p* < 0.01, Pearson’s *r*: −0.15; Fig. 2B). We chose to assess Pearson’s correlation since the goal of work was to evaluate the parameters of different models in describing the relationship between MET and *T*. Spearman’s rank correlation, being non-parametric, does not describe the *kind* of relationship (*e.g*., linear, *etc*.) between variables. Since we are interested in evaluating the way in which activity *perturbs* skin temperature, we wanted to remove the autocorrelation relationship of temperature on itself. We did so by creating a new time series based on the minute-to-minute differences in temperature (Δ*T*). We observed that Pearson’s correlation reflected a stronger linear relationship between MET to Δ*T* than of MET to *T* (*p* < 0.001, Pearson’s *r*: −0.28; Fig. 2C). When comparing the superiority of MET to *T* or MET to Δ*T* across all participants (aggregating by the median Pearson’s *r* for each participant’s set of valid windows), we observed that 88% of participants had a better *R*^2^ between MET and Δ*T* than MET and *T* (Fig. 2D).

Pearson’s correlation does not capture causal (or even directional) relationships between variables. Cross-correlation analysis revealed that a vast majority of participants (>99%) had a maximum absolute correlation between MET and Δ*T* at negative time lags, indicating that in most comparisons MET was leading changes in T (Fig. 2E). Estimates of information transfer from MET (at a lag of −1 min) to ΔT via Transfer Entropy (TE) further supported the model that activity perturbations were predictive of future Δ*T* values even when historical Δ*T* was also known (Fig. 2F). Importantly, the magnitude of TE from MET to Δ*T* negligibly decreased as age increased, with a decrease of 6.7e-5 nats per year of age. This indicates that the *timing* of the effect of MET on Δ*T* at a lag of −1 min is likely to remain consistent in older populations.

### Performance of predicting temperature deflections from activity during sleep improves when including starting and ending temperature values

**Fig. 3:**
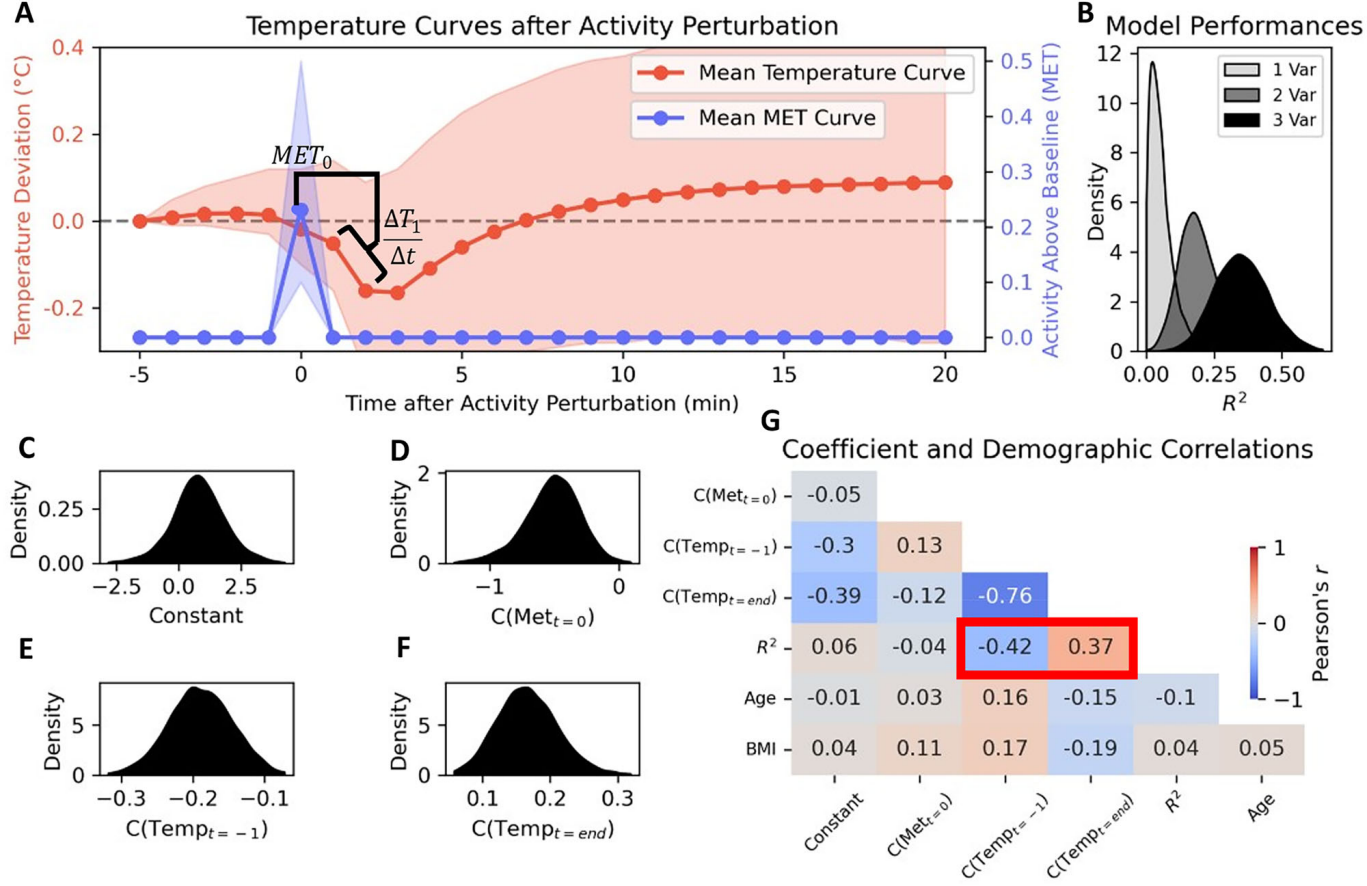
Multilinear predictions on temperature perturbations after activity event. **A** Mean temperature trajectory (red dotted line) with standard deviation (red shaded region) with mean MET trajectory (blue dotted line) with standard deviation (blue shaded region). MET is plotted for times around 0 to show the method of only selecting an isolated activity perturbation. The MET at time 0 is used to predict the change in temperature between times 1 and 2. **B** KDEs of the 1-, 2-, and 3-variable linear models in light gray, gray, and black, respectively. **C** KDE of the population fit constants. **D** KDE of the population fit coefficient for the MET magnitude at time 0. **E** KDE of the population fit for the initial temperature. **F** KDE of the population fit for the ending temperature. **G** Heatmap of the pairwise correlations between the model coefficients as well as model performance (*R*^2^), age, and BMI. Tiles are colored and annotated by the Pearson’s r between those two variables.

Given that the Transfer Entropy analysis suggested a directional relationship from MET to Δ*T* at short timescales (minutes), we were interested in quantifying the *magnitude* of that relationship on a participant-to-participant basis. To remove the potential confounding effects of consecutive non-minimum MET values on temperature trajectories, we selected 20-min windows of temperature that followed activity perturbations (See Methods - Selection of Temperature Trajectories after Activity Perturbations for more complete description of candidate temperature trajectory selection). The mean curves of MET and *T* are shown in Fig. 3A. MET values were converted to MET above baseline by subtracting 0.9 from all values (in order to remove the amplitude bias across all participants). The magnitude of MET at the time of perturbation (${{MET}}_{0}$) was used to predict the magnitude of Δ*T* between 1 and 2 min after the perturbation ($\Delta {T}_{1}$) for any candidate temperature trajectory.

Three linear models were fit to each participant’s set of trajectories, with an increasing number of variables to evaluate the impact of more variables on predictive performance. For each participant, the variables from all trajectories were concatenated in order to create personalized models. Therefore, there were 3 models for each participant, depending on which set of independent variables was used. Personalized models had 1 variable: ${{MET}}_{0}$; 2 variables: ${{MET}}_{0}$ & ${T}_{0}$ (temperature at time of perturbation); or 3 variables: ${{MET}}_{0}$ & ${T}_{0}$ & ${T}_{{End}}$ (final temperature of the temperature trajectory). The median performances (*R*^*2*^) were 0.04 (95% CI: 0.001, 0.17), 0.19 (95% CI: 0.07, 0.39), and 0.35 (95% CI: 0.17, 0.56), respectively (Fig. 3B).

Since the 3-variable model was the best performing using all trajectories from all participants, we then evaluated it in participant-level models to see how the coefficient weights related to magnitude changes in $\Delta {T}_{1}$. The majority (>99%) of participants had the same sign on their coefficients, indicating a consistent statistical relationship between each of the variables on $\Delta {T}_{1}$ magnitude. Consistent with the original global linear correlation, the median coefficient for ${{MET}}_{0}$ was −0.50 (95% CI: −1.0, −0.11), indicating its negative association with $\Delta {T}_{1}$ (Fig. 3D). The median coefficient for the starting temperature (${T}_{0}$) was −0.19 (95% CI: −0.28, −0.10), highlighting that the magnitude of the $\Delta {T}_{1}$ is likely to be larger (more strongly negative) when the steady state skin temperature of a participant is higher prior to the activity perturbation (Fig. 3E). Inversely, the median coefficient for the final temperature (${T}_{{End}}$) was 0.16 (95% CI: 0.08, 0.27). Therefore, as the final temperature increased, the total temperature decrease ($\Delta {T}_{1})$ was reduced or even reversed to a positive delta (Fig. 3F).

Ideally, in a statistically independent model, coefficients for the variables should not depend on one another in order not to confound interpretation. We observed that the coefficients for ${T}_{0}$ and ${T}_{{End}}$ had a strong linear relationship with each other with a Pearson’s *r* = −0.76 (Fig. 3G). Furthermore, we saw that the *performance* of these models is negatively correlated to ${T}_{0}$ (Pearson’s *r* = −0.42) and positively correlated to ${T}_{{End}}$ (Pearson’s *r* = 0.37). These observations on the qualities of all participant-level multilinear models support the conclusion that these coefficients or relationships may be dependent and non-linear. While the inclusion of two correlated features (${T}_{0}$ and ${T}_{{End}}$) may not be ideal for a multilinear model, they would both be necessary to include for a model aiming to parameterize temperature dynamics, since temperature changes proportional to the starting and steady state temperature of a system. This led us to design a state-dependent model of skin temperature changes that accounts for each trajectory’s variability in time, dependent on the AP magnitude and the temperature distance from steady state (Eq. 4).

### A mechanistic model of activity and temperature effects accounts for substantially more variance in temperature perturbations than a multilinear model

**Fig. 4:**
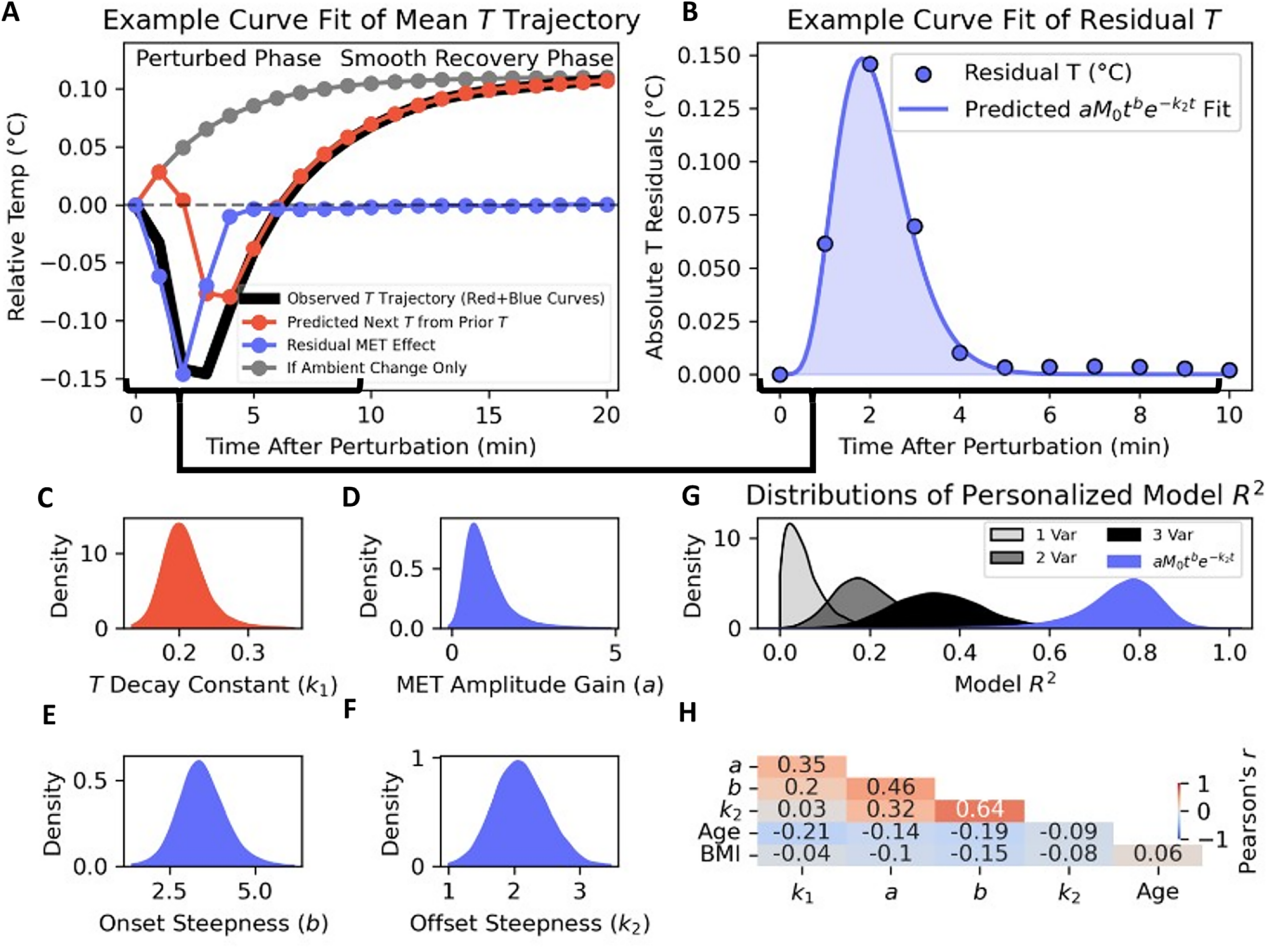
Residual temperature curves and their parameters after removing state-dependent heating dynamics. **A** Temperature curves: Observed mean temperature trajectory (black solid curve)as an example trajectory, its state-dependent (minute-by-minute) prediction of next temperature based on current temperature distance from final temperature (red dotted curve), residual temperature trajectoryafter removing the (red) temperature prediction (blue dotted curve), and approximated temperature curve if it were the case that there was only an ambient temperature change and no activity effect (gray dotted curve). **B** Example absolute residual MET effect from A (blue scatter plot) as well as the continuous curve when fitting the time-dependent decaying exponential to it (blue solid curve). **C** KDE of the population median temperature decay constants from the smooth recovery phase of the curves. **D** KDE of the population median MET amplitude gain from the perturbed phase of the curves. **E** KDE of the population median onset steepness from the Perturbed Phase of the curves. **F** KDE of the population median offset steepness from the Perturbed Phase of the curves. **G** KDEs from Figure 3B reused here along with the KDE for the *R*^2^ from using the time-dependent decaying exponential to predict the entire MET effect curve. **H** Heatmap of the pairwise correlations between the model coefficients as well as age. Tiles are colored and annotated by the Pearson’s *r*.

If it were the case that such a mechanistic model followed first-order heating dynamics, where temperature attempts to return to a new steady state value after perturbation, then we would anticipate that the observed temperature curves change at a rate relative to how far the current temperature is from the final temperature (Fig. 4A, gray dotted line). It is important to note that a stepwise change in ambient temperature would appear as the gray dotted line in Fig. 4A. However, the observed skin temperature data clearly show a perturbation phase, suggesting a more complex response – at least at the beginning of the temperature trajectory. After about 6 min, the temperature trajectory dynamics appear to follow a smooth recovery phase back to a new steady state.

A single stepwise negative change in ambient temperature (such as movement of the hand in/out of a microclimate (*e.g*., covers/blanket)) would not be able cause the negative perturbation in the temperature trajectory unless there was a subsequent *different stepwise change* in ambient temperature again to cause it to heat back up. We instead propose that it is more likely that it is the continuous effect of the activity perturbation dynamics that is causing a transient cooling effect in finger skin temperature. We tested this hypothesis by first evaluating the temperature trajectory dynamics in the second half of each trajectory (minutes 10–20) and estimating the decay constant (${k}_{1}$) when the residual temperature effect was likely negligible. We then predicted the entire temperature trajectory (starting at index 0) as if it were a state-space model — we predicted each subsequent *T* value based on the current observed value’s distance from the steady state temperature and multiplied it by ${k}_{1}$ (Fig. 4A, red dotted line; Eq. 4: ${\Delta T}_{t}={k}_{1}\cdot ({T}_{{end}}-{T}_{t})$, see Methods - Modeling of Activity Perturbation Trajectory from Temperature Recovery Dynamics for more detail on model assumptions). By subtracting the predicted temperature trajectory from the observed temperature trajectory, we were left with the unexplained, residual temperature data that we attributed to the dynamics of the activity perturbation (Fig. 4A, blue dotted line).

The residual temperature signals were not random noise (Fig. 4B). The trajectory of the residual data appeared to follow a time-dependent exponentia l— brief, high-amplitude increase from zero followed by an exponential decay back to zero as time approaches infinity. An explicit fitting of such a model against the residual mean ${\epsilon }_{T}\left(t\right)$ data revealed an *R*^2^ = 0.96 (Eq. 5: ${\epsilon }_{T}\left(t\right)=a\cdot {M}_{0}\cdot {t}^{b}{e}^{-{k}_{2}t}$, see Methods - Modeling of Activity Perturbation Trajectory from Temperature Recovery Dynamics for more detail on model assumptions). There were no significant differences in model performances when separating by sex (Supplementary Fig. 2). Confident in the population-level fit, we continued to fit the Eq. 5 model to each residual temperature trajectory after removing the steady-state effects of Eq. 4 from each temperature trajectory.

The result was a personalized model of temperature trajectories after activity perturbations for each individual, with each coefficient reflecting the dynamics of entire curves, instead of only reflecting instantaneous linear correlations (Fig. 4C–F). More importantly, the performance predicting the entire temperature trajectory (Eq. 5) was substantially larger than that of the 3-variable multilinear model, achieving a median *R*^2^ = 0.77 (95% CI: 0.52, 0.89; Fig. 4G), with the same number of parameters. We observed strong correlations between the residual temperature trajectory parameters but only negligible to moderate correlations between those parameters to the temperature recovery decay constant, *k*_*1*_ (Fig. 4H). We also observed significant, small correlations between each of the parameters and age. Interestingly, the maximum explained variance from body mass index (BMI) for any of the parameters was 2.3% (Pearson’s *r*: −0.15, *R*^*2*^*: 0.023)* for the onset steepness, $b$, indicating a negligible relationship between the model parameters and reported BMI in this work.

### More representative physiological models have parameters that better reflect cardiovascular perfusion deficits

While the model performance provided confidence that we were sufficiently capturing the temperature dynamics, the fit coefficients for each trajectory had the potential to provide insight into physiological differences between individuals. We sought to evaluate how each of the dynamical parameters separated cohorts with conditions associated with cardiac and cardiovascular deficits against a cohort with no reported conditions (see Methods - Comparison of Dynamical Model Coefficients to Reported Conditions for more details on cohort selection and permutation testing for effect size distributions). We also evaluated whether the dynamical model coefficients outperformed the multilinear model coefficients to determine if a simpler linear model would be sufficient for a similar magnitude of cohort separations.

**Fig. 5:**
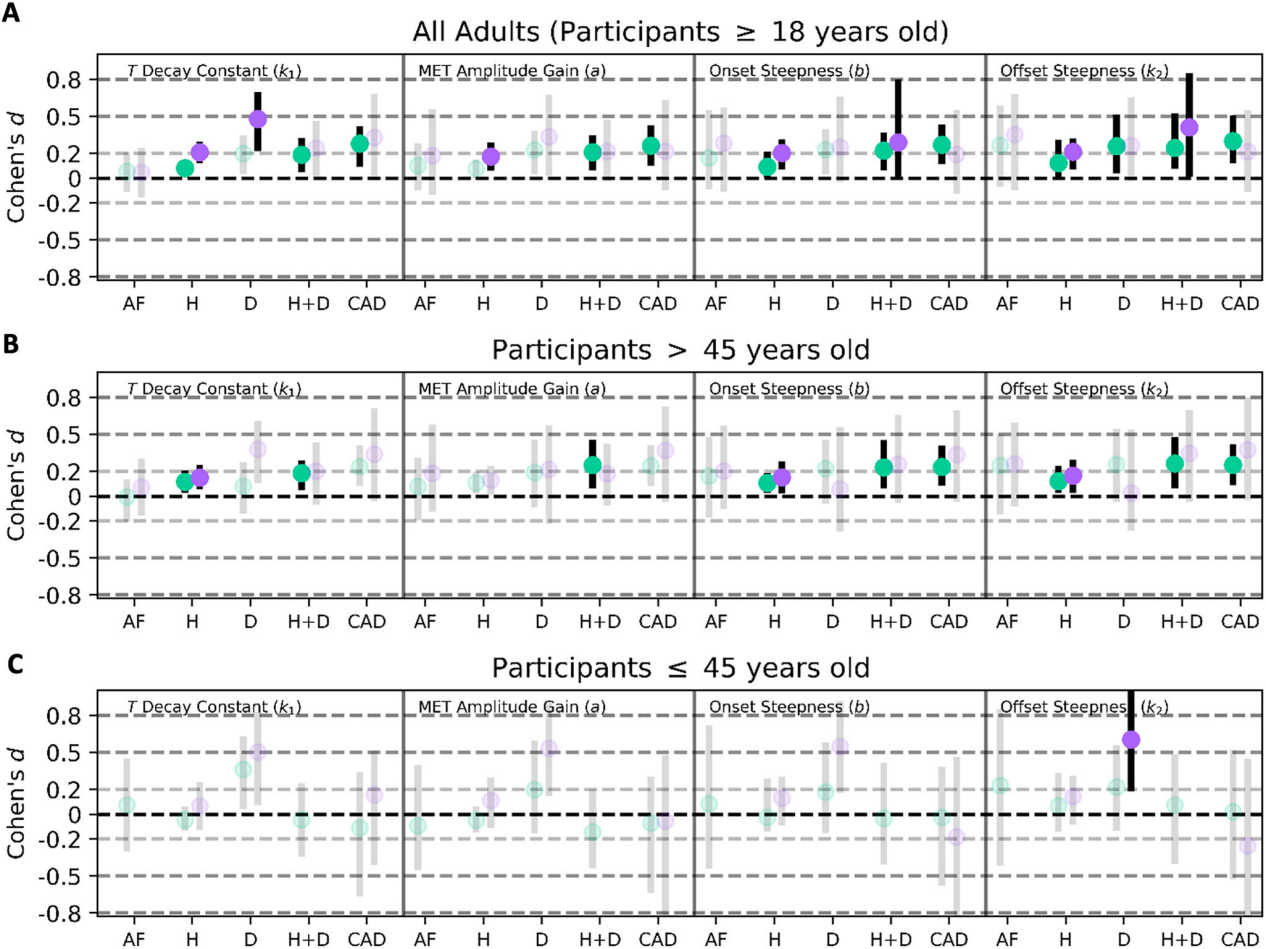
Cohort separability by dynamical model parameters across multiple cardiovascular diseases. Median Cohen’s *d* values (circular scatterplot markers) of male (cyan) and female (purple) model coefficients based on comparing a sex-matched cohort with no reported conditions against cohorts with reported Atrial Fibrillation (AF), Hypertension (H), Diabetes Mellitus (D), both H and D (H+D), and Coronary Artery Disease (CAD). The black lines through each median circular marker reflect the 2.5th percentile (lowest end) and 97.5th percentile (highest end) of the distribution of odds-ratios when comparing the cohort with a condition against a size-matched random selection from the control cohort (with replacement) 1000 times. Markers that are solid (not transparent) reflect that the original Mann–Whitney U test to compare cohorts was significant after Bonferroni correction *and* the 2.5th and 97.5th percentiles of the distribution of odds-ratios are either both *above* 0 (the parameters are greater in the control cohort) or are both *below* 0 (the parameters are lower in the control cohort). Each subplot is then an age-separated cohort of either the original multilinear model parameters (left subplots) or the time-dependent model (right subplots). **A** Time-dependent model parameters from participants who were at least 18 years old. **B** Time-dependent model parameters from participants who were at least 46 years old. **C** Time-dependent model parameters from participants who were at most 45 years old.

For the coefficients fit from the multilinear models, there were no significant effects out of the 120 comparisons (Supplementary Fig. 3A–C). The median parameters for dynamical models fit to participants were much better at separating cohorts with cardiac and cardiovascular conditions. There were 33 significant (after Bonferroni correction) effects out of the 120 possible comparisons. For males in all ages, CAD compared to control had the greatest effect size across all dynamical parameters (Median Cohen’s *d* of 0.26, 0.26, 0.27, and 0.30 for ${k}_{1}$, $a$, $b$, and ${k}_{2}$, respectively; Fig. 5A). For females in all ages, the largest effect sizes were observed in groups with diabetes (D) and the groups with diabetes comorbid with hypertension (H+D) compared to control. However, when separating by age in females, the greatest effect sizes by feature were seen in groups with hypertension (H) for the ${k}_{1}$ and ${k}_{2}$ parameters (Median Cohen’s *d* of 0.15 and 0.17, respectively; Fig. 5B). Furthermore, there was a moderate effect size in the *younger* D cohorts for female ${k}_{2}$ (Median Cohen’s *d* of 0.61; Fig. 5C).

While these dynamical parameters appeared to separate the cohorts with cardiac and cardiovascular-associated conditions of interest against control cohorts, each of these conditions might have contributed independent shifts to the parameter distributions. The effects of sex and age might have been heterogeneous across the parameters as well. We fit four multilinear models to the parameters, one model for each parameter, to identify any differences in the weight contributions across these conditions that might have explained the different effect sizes observed in Fig. 5.

A significant sex effect was present only in the ${k}_{1}$ parameter (*p* < 0.001, −0.21 (95% CI: −0.25, −0.18)) but not in the other parameters. Sex also had the greatest β weight out of all other variables for ${k}_{1}$ (Table 1). There were significant effects of age across all parameters, all with relatively similar β weights (*p* < 0.001 for all parameters; ${k}_{1}$: −0.11 (95% CI: −0.13, −0.10); $a$: −0.09 (95% CI: −0.11, −0.07); $b$: −0.12 (95% CI: −0.12, −0.10); ${k}_{2}$: −0.12 (95% CI: −0.14, −0.10)). There were significant *β* weights for Atrial Fibrillation only for the $b$ (*p* < 0.05, −0.14 (95% CI: −0.27, −0.02)), and ${k}_{2}$ (*p* < 0.05, −0.14 (95% CI: −0.27, −0.02)) parameters. There were significant *β* weights for Hypertension for the $a$ (*p* < 0.05, −0.05 (95% CI: −0.09, 0.00)), $b$ (*p* < 0.001, −0.13 (95% CI: −0.17, −0.08)), and ${k}_{2}$ (*p* < 0.001, −0.15 (95% CI: −0.19, −0.10)) parameters. There were significant *β* weights for Diabetes for the ${k}_{1}\,$(*p* < 0.001, −0.16 (95% CI: −0.25, −0.07)), $b$ (*p* < 0.01, −0.15 (95% CI: −0.24, −0.06)), and ${k}_{2}$ (*p* < 0.001, −0.27 (95% CI: −0.36, −0.18)) parameters. Finally, CAD had a significant *β* weight for ${k}_{1}$ (*p* < 0.05, −0.14 (95% CI: −0.27, −0.01)).

**Table 1:** Multilinear model performance and coefficient weights when fitting multiple potentially comorbid conditions with sex and age to the time-dependent model parameters

	*k*_*1*_ *(R*^*2*^ = *0.03)*		*a (R*^*2*^ = *0.01)*		*b (R*^*2*^ = *0.02)*		*k*_*2*_ *(R*^*2*^ = *0.03)*	
	*β [95% CI]*	*P* > *|t* |	*β [95% CI]*	*P* > *|t* |	*β [95% CI]*	*P* > *|t* |	*β [95% CI]*	*P* > *|t* |
*Constant*	**0.10 [.07,** **.12]**	**<0.001**	0.01 [−0.01, 0.04]	> 0.05	**0.04 [0.02, 0.06]**	**<0.01**	**0.03 [0.00, 0.05]**	**<0.05**
*Sex*	**−0.21 [−0.25, −0.18]**	**<0.001**	−0.00 [−0.04, 0.03]	> 0.05	−0.02 [−0.05, 0.01]	>0.05	0.03 [−0.00, 0.07]	>0.05
*Age*	**−0.11 [−0.13, −0.10]**	**<0.001**	**−0.09 [−0.11, −0.07]**	**< 0.001**	**−0.12 [−0.14, −0.10]**	**<0.001**	**−0.12 [−0.14, −0.10]**	**<0.001**
*Atrial Fibrillation*	0.06 [−0.06, 0.19]	>0.05	−0.01 [−0.14, 0.11]	> 0.05	**−0.14 [−0.27, −0.02]**	**<0.05**	**−0.14 [−0.27, −0.02]**	**<0.05**
*Hypertension*	−0.03 [−0.08, 0.01]	>0.05	**−0.05 [−0.09, −0.00]**	**< 0.05**	**−0.13 [−0.17, −0.08]**	**<0.001**	**−0.15 [−0.19, −0.10]**	**<0.001**
*Diabetes*	**−0.16 [−0.25, −0.07]**	**<0.001**	−0.06 [−0.16, 0.03]	> 0.05	**−0.15 [−0.24, −0.06]**	**<0.01**	**−0.27 [−0.36, −0.18]**	**<0.001**
*Coronary Artery Disease*	**−0.14 [−0.27, −0.01]**	**<0.05**	−0.05 [−0.19, 0.08]	>0.05	−0.11 [−0.24, 0.02]	>0.05	−0.09 [−0.22, 0.04]	>0.05

Bolded weights are significant.

## Discussion

In this work, we find that models designed to explicitly account for the dynamics of skin temperature can surface physiologically relevant parameters that distinguish cohorts with conditions linked to deficiencies in peripheral blood perfusion. We began by identifying the limitations of assuming a purely statistical, time-invariant relationship between sleep activity and skin temperature. Wearables-derived temperature and activity signals do not follow the IID assumptions required for standard correlation analysis. While Pearson’s correlation captures linear covariance, it does not account for time-dependent effects. Skin temperature fluctuates even when MET is minimal^52^, suggesting that a linear statistical relationship alone cannot explain its variation.

To explore the directional effects between activity and temperature, we applied linear temporal (cross-correlation) and information-theoretic (Transfer Entropy) analyzes. These methods indicated that MET has a minute-level directional effect on *T*, though the reverse effect — *T* influencing MET — is primarily observed over circadian timescales^53–55^ (hours) rather than minutes. When analyzing isolated activity perturbations (APs) and their effect on temperature change 1 min ahead ($\Delta {T}_{1}$), we found that MET alone explained only negligible to small variance across participants. However, including the starting temperature (${T}_{0}$) and ending temperature (${T}_{{End}}$) of a temperature trajectory significantly improved multilinear model performance. Notably, ${T}_{0}$ and ${T}_{{End}}$ were highly colinear, suggesting that activity perturbations act as additional effects on top of the inherent heating dynamics of skin temperature as it approaches a steady-state value.

Building on this, we developed a dynamical model under the assumption that temperature trajectories follow Newton’s Law of Heating in a state-dependent manner. By fitting a time-dependent decaying exponential to the residual activity effect, we demonstrated that this trajectory-based model explained a large proportion of variance while maintaining the same number of parameters as the multilinear model. Crucially, evaluating the clinical relevance of these model parameters revealed moderate to large effect sizes when comparing cohorts with reported cardiac and cardiovascular conditions to a control cohort without reported conditions, with additional effects of age and sex. Importantly, these effect sizes were not observed in the coefficients of the lower-performing multilinear model, which predicted only an instantaneous activity effect rather than the full activity effect curve. This work supports efforts in identifying *dynamical biomarkers* — biomarkers that capture the trajectory of physiological signals over time rather than relying solely on statistical aggregates or moments. While average skin temperature may correlate with certain acute and chronic conditions, understanding the rules governing its minute-to-minute changes provides deeper insight into the mechanisms that regulate it.

We provided evidence that the parameterization of skin temperature dynamics after a transient activity event during sleep carried information related to whether a participant had a reported cardiovascular condition. We also showed that the magnitudes of these effects are age- and sex-dependent even when accounting for the presence of multiple reported conditions. We believe that the temperature decay constant, ${k}_{1}$, reflects a combination of thermal inertia from BMI and body surface area (BSA), ambient temperature, and the resting vascular tone after a perturbation since it reflects the dynamics of the temperature trajectory during the smooth recovery phase (in an ideal case, the effects of BMI and BSA are constant, and ambient temperature does not change). We would anticipate seeing sex differences in this variable when accounting for age due to different body mass to BSA ratios, as well as differences in local temperature perceptions. The MET amplitude gain, $a$, could be interpreted as the participant-specific effect that reflects how sensitive the vasoconstrictive response is to a given magnitude of transient activity. The onset steepness, $b$, may indicate the vasoconstrictive elasticity in response to the vasoconstrictive response while the offset steepness, ${k}_{2}$, would be the refractory or vasodilative response after perturbation. We hypothesize that the $b$ and ${k}_{2}$ parameters are most associated with conditions that are highly comorbid with arteriosclerosis since they would approximate the elasticity of the peripheral vasculature.

There are several limitations of this modeling approach that we would ameliorate in a validation study to evaluate if these participant-specific coefficients are truly associated with perfusion deficiencies or arteriosclerosis: (1) Due to the uncontrolled nature of the data, the ambient temperature when a temperature trajectory occurs is not known. Ambient temperature directly affects vascular tone as well as the rate of heat transfer from the skin to the environment. However, we believe it is unlikely that ambient temperature differences would explain the effect sizes observed between the cohorts, since it would require that a significant proportion of those groups would select for sleeping in a cooler environment. (2) The approximation of activity, MET, acts as an ordinal variable for low values of activity due to its step-size of 0.1 and its lowest and 99th percentile of values being 0.0 and 0.6 above the lower limit of detection (0.9 MET), respectively. High frequency accelerometry data may be a better approximate of activity perturbation amplitude due to its finer time and amplitude resolution. (3) We do not know for how long the participants in the cohort have had their conditions. If the parameters of our proposed model reflect arterial stiffness or perfusion deficiencies, age is likely a secondary covariate to a participant’s *time since diagnosis* for conditions in which arteriosclerosis is comorbid. (4) The trajectory of the activity effect is not mechanistically a function of time—we have only chosen to model it as such for this work. It is much more likely that the trajectory is *state-*dependent. That is, the entire curve is more dependent on the initial vascular tone, the magnitude of the activity perturbation, as well as the state of the lumen radius *at each moment in time*. Despite these limitations, we believe that this work serves as a proof of concept toward using wearables-derived skin temperature data to identify changes in cardiovascular responses and should be of interest to the wearables community as a whole in regard to the role of mathematical modeling of physiological data to surface physiological data that is difficult to ascertain from statistical- or magnitude- based analyzes alone.

## Methods

### Wearable device and questionnaire data collection

All data were part of the TemPredict Study and were collected from January to November, 2020^56^. The dataset includes physiological data generated using the wearable device Oura Ring, as well as survey data such as self-reported sex, age, race, and ethnicity. All participants wore the Oura Ring Gen2 (Oura Health Oy, Oulu, Finland), a commercial wireless device worn on the finger that, among other things, contains 2 sensors relevant here: (1) negative coefficient thermistor (resolution of 0.07 °C) to detect distal skin temperature (*T*), and (2) a tri-axial accelerometer to measure activity (metabolic equivalents, MET, resolution of 60 s)^11^. All data were wirelessly synced via Bluetooth from the ring to the user’s smartphone when the Oura App was in use. Data from the app were then sent by the app to Oura’s cloud architecture, from which we received a one-time data push from their secure Amazon storage (S3) to our S3 cloud storage located on San Diego Supercomputer infrastructure^57^.

In addition to the wearable data, participants had the option to answer a questionnaire related to chronic illness via the app. Additionally, demographic questionnaire data included age in years (“What is your age?”) and self-reported sex (“What is your biological sex?”). A question related to illness asked, “Have you ever been told by a healthcare professional that you have, or have been treated for, any of the following conditions (in the past or currently)?” with options: high blood pressure or hypertension, diabetes, CAD or angina, a heart attack, congestive heart failure, stroke or transient ischemic attack, atrial fibrillation, sleep apnea, COPD (emphysema, chronic bronchitis), asthma requiring regular inhaler use, cancer, anemia or other blood disorder, immunodeficiency including HIV, or other respiratory issues and “None of these”. The latter option indicates the participant reports not having been diagnosed with any of the listed conditions.

### Ethics approval and consent to participate

The University of California San Francisco (UCSF) Institutional Review Board (IRB, IRB# 20-30408) and the U.S. Department of Defense (DOD) Human Research Protections Office (HRPO, HRPO# E01877.1a) approved of all study activities, and all research was performed in accordance with relevant guidelines and regulations and the Declaration of Helsinki. All participants provided informed electronic consent. We did not compensate participants for participation.

### Selection of participant data for activity and temperature coupling analysis

Participants with survey responses regarding health conditions were considered eligible for analysis. We selected windows of participant *T* and MET data where there were at least 300 contiguous samples (5 h) of data that were generated during sleep, based on Oura’s proprietary algorithmically defining sleep windows. The contiguous 5-h duration was chosen as it is more likely to represent a window of long-term sleep instead of a transient nap. The end of the window is based on the first “awake” sample after the window; therefore, the minimum length of each window is 300 samples.

### Calculation of global relationships – Pearson’s correlation and transfer entropy

Global relationships between variables were first performed to find the likely associative and directional of causal factors for dynamical modeling. For each valid window of sleep, two Pearson’s correlation analyzes were performed: (1) the linear correlation of MET and distal skin temperature and (2) the linear correlation of MET and the *minute-to-minute change* in skin temperature (Δ*T*). The median test statistics (Pearson’s *r*) were calculated for each analysis for each participant to ensure IID comparisons since there were multiple nights per participant.

To evaluate potential causality between temperature and MET, Transfer Entropy (TE) was chosen as a non-parametric statistic measuring the amount of *directed* transfer of information between two processes^58^. TE quantifies the information that can be learned about a future value in a process, Y, if the past information of a source process, X, and past information of Y are known. Unlike Pearson’s correlation, TE is an asymmetrical measurement—that is, the TE from MET to ΔT is not equal to the TE from Δ*T* to MET if one of those signals disproportionately carries information about future values of the other. It is also robust to nonlinear relationships between variables.

### Selection of temperature trajectories after activity perturbations

**Fig. 6:**
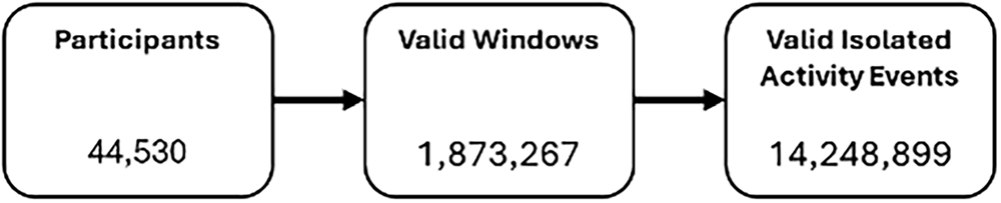
Activity event quantity from cohort. Flowchart reflecting the number of eligible participants, the total number of valid nights from all participants, and the total number of valid isolated activity events from those nights from all participants.

For each sleep window, we first identified activity events during sleep as the samples in which a non-minimum MET value was measured (Note: the Oura data structure for MET has the minimum valid value set to 0.9). An activity event was classified as an Activity Perturbation (AP) if no other activity events occurred during the 20 min before or after it. Temperature data were then taken from 1 min prior to 20 min post an AP. This increased the likelihood that any such candidate Temperature Trajectory (TT) and activity perturbation occurred during a time of a participant being inactive during sleep, as well as at a temperature steady state. The number of valid participants, nights, and isolated MET events/temperature trajectories are shown in Fig. 6.

### Activity linear univariate and linear multivariate models of temperature perturbation

Three linear models were created using ordinary least squares regression (*Python v3.11*.5; *statsmodels v0.11*.0) to evaluate the relationship between MET amplitude, starting temperature (*T*_0_), and ending temperature (*T*_end_) (independent variables) on the change in temperature 1 min after the AP (Δ*T*_1_ – dependent variable). Each independent variable was incrementally added to evaluate the performance changes of a linear model upon the inclusion of more variables. *T*_end_ was included as a variable since, at the end of a trajectory, it is an approximation of steady state temperature when the rate of change of temperature is negligible. Furthermore, steady state temperature can change throughout the night due to changes in hand location, making it necessary to include it in the model as a covariate. As steady state temperature is an important factor in heating dynamics, we wanted to evaluate its inclusion under assumptions of linearity prior to including it in nonlinear models. Those three models were fit to data from each participant, leading to personalized linear predictions/models for each individual.

1 $$\Delta {T}_{1}={{{\beta }_{0}+\beta }_{1}{MET}}_{0}$$

2 $$\Delta {T}_{1}={{{\beta }_{0}+\beta }_{1}{MET}}_{0}+{\beta }_{2}{T}_{0}$$

3 $$\Delta {T}_{1}={{{\beta }_{0}+\beta }_{1}{MET}}_{0}+{\beta }_{2}{T}_{0}+{\beta }_{3}{T}_{{end}}$$

#### Modeling of activity perturbation trajectory from temperature recovery dynamics

Two assumptions were made to evaluate the dynamics of temperature trajectories after activity perturbations: (1) skin temperature follows Newton’s Law of Heating (the rate of skin temperature change back to a steady state value is proportional to how far skin temperature is from that steady state value) and (2) the effect magnitude of activity on skin temperature change after an AP decays to 0 faster than skin temperature returns to steady state. This assumption partitions each temperature trajectory into two portions. The first portion is the Perturbed Phase, where skin temperature dynamics do not appear to follow the expected heating dynamics; the second portion is the Smooth Recovery Phase, where skin temperature dynamics *do* appear to follow the expected heating dynamics

4 $${\Delta T}_{t}={k}_{1}\cdot ({T}_{{end}}-{T}_{t})$$

where ${T}_{{end}}-{T}_{t}$ is the skin temperature difference between the steady state value and the current skin temperature value, ${\varDelta T}_{t}$ is the rate of change of skin temperature value, and ${k}_{1}$ is the decay constant that reflects the magnitude of effect that ${T}_{{end}}-{T}_{t}$ has on ${\Delta T}_{t}$. The decay constant, $k$, was first calculated by fitting the above equation to the temperature data starting 10 min *after* the activity perturbation (during what is likely the Smooth Recovery Phase. Note—this is chosen heuristically based on the observed trajectory data since 95% of the trajectories had the best Eq. 4 model fit starting at or after 10 min (Supplementary Fig. 1). That $k$ was then used to predict the ${\varDelta T}_{t}$ at each minute after the AP based on the *observed* skin temperature data sampled at that minute. The difference between the observed temperature trajectory and the *predicted* temperature trajectory from Eq. 4 can then be considered the unaccounted for, residual temperature dynamics that are likely the effect of transient activity on the Perturbed Phase of the trajectory.

**Fig. 7:**
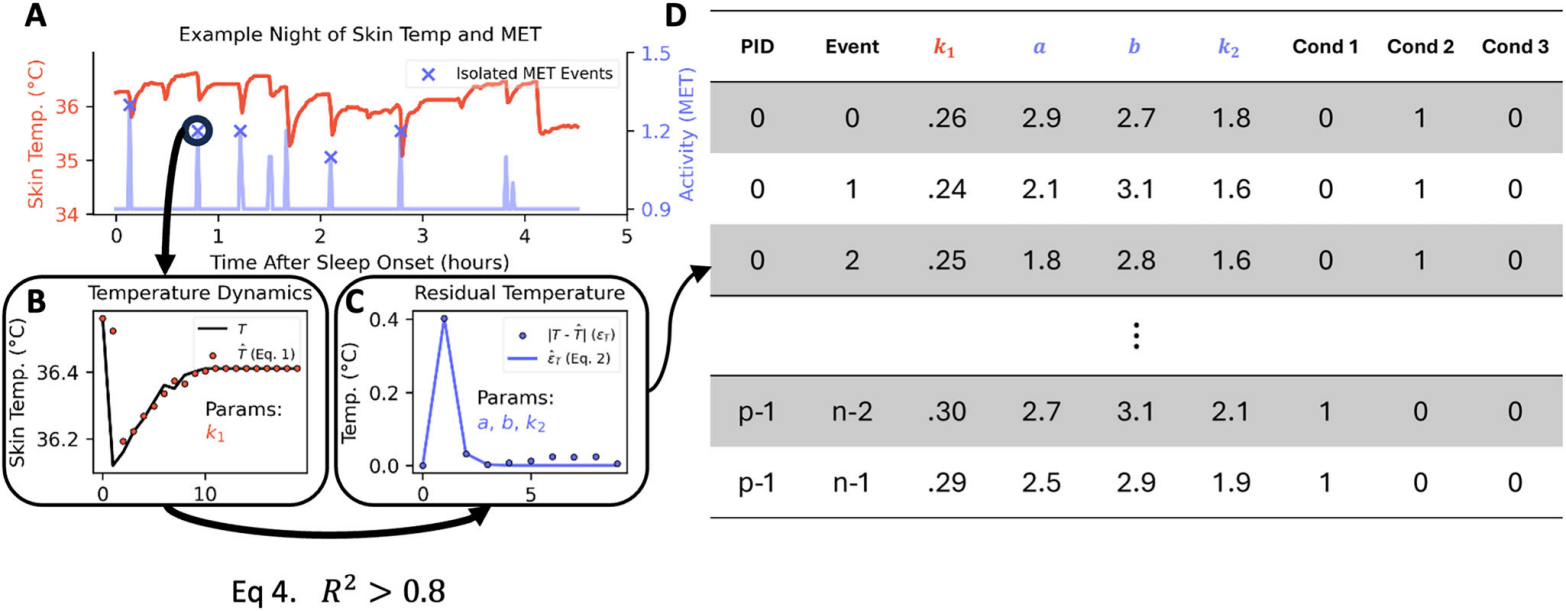
Example computational workflow of processing isolated activity (MET) events. **A** An example night of skin temperature (red) and activity (MET, blue) signals. An isolated MET event is one in which there is a non-minimum MET value that does not have any other non-minimum MET values both 20 min before and 20 min after it (denoted with a blue ‘X’). 20-min segments of temperature data (starting at each isolated MET event and for 20 min) are then chosen as valid temperature trajectories for analysis. **B** A single example of a temperature trajectory with true temperature (black) and estimated temperature from first-order state-dependent effects based on Eq. 4 (red scatterplot). The first-order dynamics (*k*_1_) are approximated from Eq. 4 using scipy.curve_fit by integrating predicted temperature values. Valid temperature trajectories are ones in which the predicted first-order dynamics fit the observed data very well (*R*^2^ > 0.8). **C** The absolute residual temperature signal (blue) after removing the state-dependent effects predicted from Eq. 4. The parameters of the residual dynamics (a, b, k_2_) are approximated from Eq. 5 using scipy.curve_fit. **D** The dynamical coefficients from each valid temperature trajectory are appended to a dataframe keeping track of which participant ID (PID) had which estimated coefficients. Statistical comparisons and effect sizes are then calculated by taking the median coefficient value for an individual if they have a condition of interest (hypertension, diabetes, etc.) being compared to a control cohort.

The trajectory of the residual temperature signal (${\epsilon }_{T}$) after removing the state-dependent heating effects appeared to follow the dynamics of a time-dependent exponential decay:

5 $${\epsilon }_{T}\left(t\right)=a\cdot {M}_{0}\cdot {t}^{b}{e}^{-{k}_{2}t}$$

where ${\epsilon }_{T}\left(t\right)$ is the residual temperature signal as a function of time ($t$), ${M}_{0}$ is MET at the time of the activity perturbation ($t=0$), $a$ is the amplitude modulation of the function, $b$ is the exponential effect of time, and ${k}_{2}$ is the rate of decay of ${\epsilon }_{T}\left(t\right)$ to 0. The model of ${\epsilon }_{T}\left(t\right)$ was only fit to residual temperature signal if the corresponding fit of the temperature trajectory to Eq. 4 had a coefficient of determination, or ${R}^{2},\ge 0.8$ to ensure the $k$ from the temperature fit was approximated from a well-fit model. A summary of the implementation of both equations to get the parameters associated with conditions for each individual can be found in Fig. 7.

6 $${R}^{2} > 0.8$$

#### Comparison of dynamical model coefficients to reported conditions

**Fig. 8:**
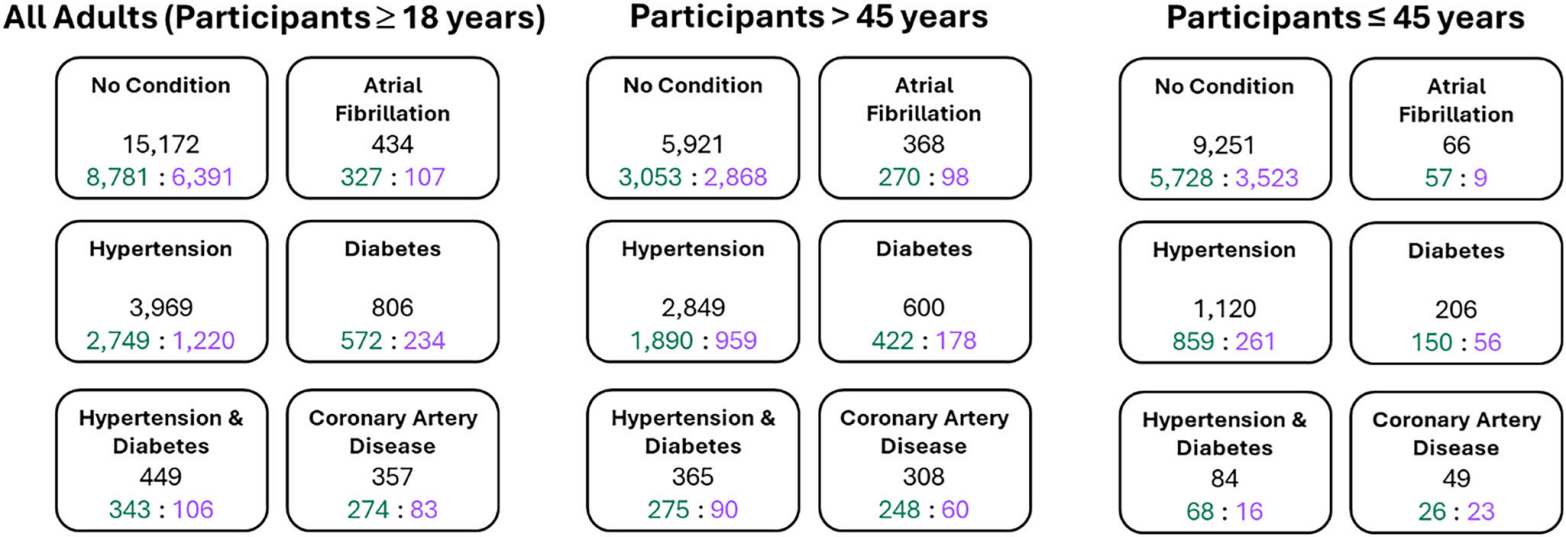
Cohort size across cardiovascular conditions. Cohort sizes when splitting by condition (each rectangle), age (left to right), and sex (Male: cyan; Female: purple).

The median coefficient values of the trajectory models, as well as the multilinear models for each individual, were calculated to maintain IID comparisons. To test the hypothesis that temperature trajectory dynamics reflect information about peripheral blood perfusion to the finger, we first separated the participants into the following cohorts depending on their reported conditions from survey data: None (No Conditions), Atrial Fibrillation (AF), Hypertension without Diabetes Mellitus (H), Diabetes Mellitus without Hypertension (D), the combination of Hypertension and Diabetes Mellitus (H+D), and CAD. We also further separated those cohorts based on reported sex^59^ (Male/Female) and age. As the average age of onset is similar for diabetes (US average age of onset 47–52, depending on race/ethnicity^60^) and hypertension (mean diagnosis age = 46^61^), we selected 45 years as the cutoff between a lower and higher aged cohort. Cohort sizes for each demographic split can be seen in Fig. 8.

We compared median temperature trajectory coefficients of cohorts with reported conditions to those of their respective sex/age-matched No Condition cohorts using Mann-Whitney U tests ($\alpha =4.1{e}^{-4}$ after Bonferroni correction with 120 tests). Within-participant coefficients for the Activity Effect were only included if the fit to Eq. 5 had a coefficient of determination, or ${R}^{2},\ge 0.5$. Effect sizes of the dynamical model coefficients (${k}_{1},\,{k}_{2},{a},{b}$) were reported as Cohen’s *d*, an estimate of effect size, for each comparison with reported health conditions (vs. no conditions, with separate comparisons for each condition) as the outcome. Separate models were run for each combination of age group and sex. A Cohen’s *d* greater than 0 reflects the control cohort having greater values for a given coefficient, whereas a Cohen’s *d* less than 0 reflects the control cohort having lower values for a given coefficient. We then created a unique multilinear regression model for each of the dynamical model coefficients (${k}_{1},\,{k}_{2},{a},{b}$) with the following independent variables to identify their relative weights: age, sex, AF, H, D, and CAD.

## Supplementary information

Supplementary information

## Data Availability

Oura’s data use policy does not permit us to make wearable device data (collected via the Oura Ring) available to third parties. We can make self-report data available; please contact Ashley E. Mason and Benjamin L. Smarr to obtain an application to obtain these data.
