# Supplementary material for: Wearable-derived skin temperature dynamics during sleep reveal cardiovascular perfusion deficits through mechanistic modeling
Source: NPJ Digit Med. 2026 Apr 22;9:464. doi: 10.1038/s41746-026-02633-2 (PMC13272961; doi:10.1038/s41746-026-02633-2)
Supplement: Supplementary file 1 — Supplementary information [file 41746_2026_2633_MOESM1_ESM.pdf]

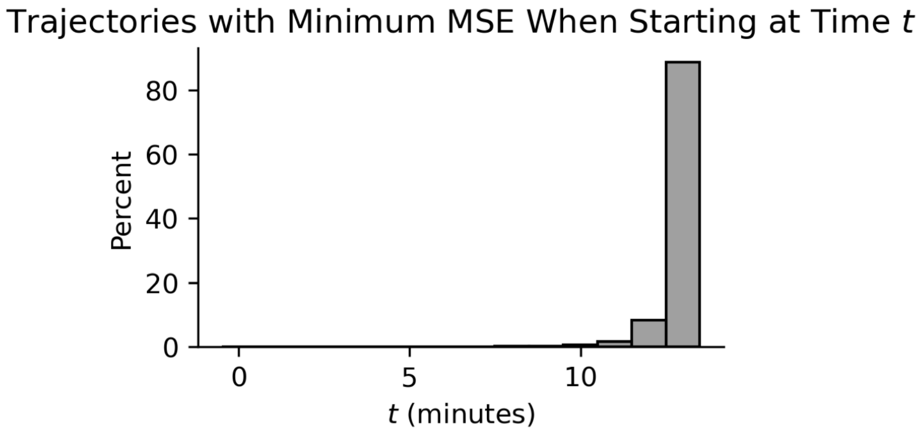

***Supplementary Figure 1*. Activity perturbation effects are likely less than 10 minutes**. The percentage of temperature trajectories in which the minimum mean squared error (MSE) when fitting for the first order dynamics of Equation 4 is met when starting with temperature at time *t* of the trajectory. For example, over 80% of temperature trajectories have a minimum MSE for first order dynamics when starting at timepoint 13 out of 20.

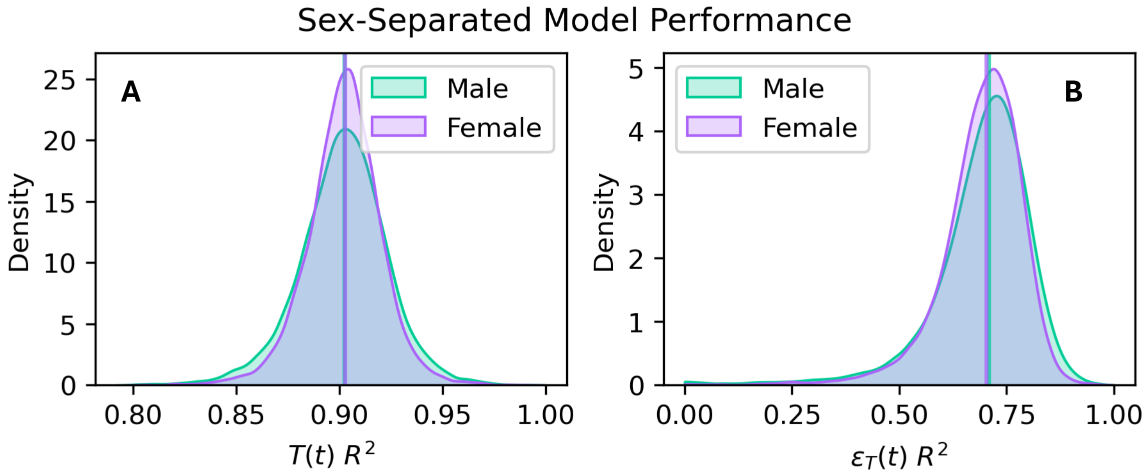

***Supplementary Figure 2.* Model performance is not significantly different between sexes**. **A.** Coefficient of determination (R^2) differences when fitting to temperature trajectories' first-order dynamics with Equation 4, separated by males (cyan) and females (purple). **B.** R^2 differences when fitting to *residual* temperature signal dynamics with Equation 5, separated by males (cyan) and females (purple). Median R^2 for males and females in both subplots are denoted with vertical lines of matching colors.

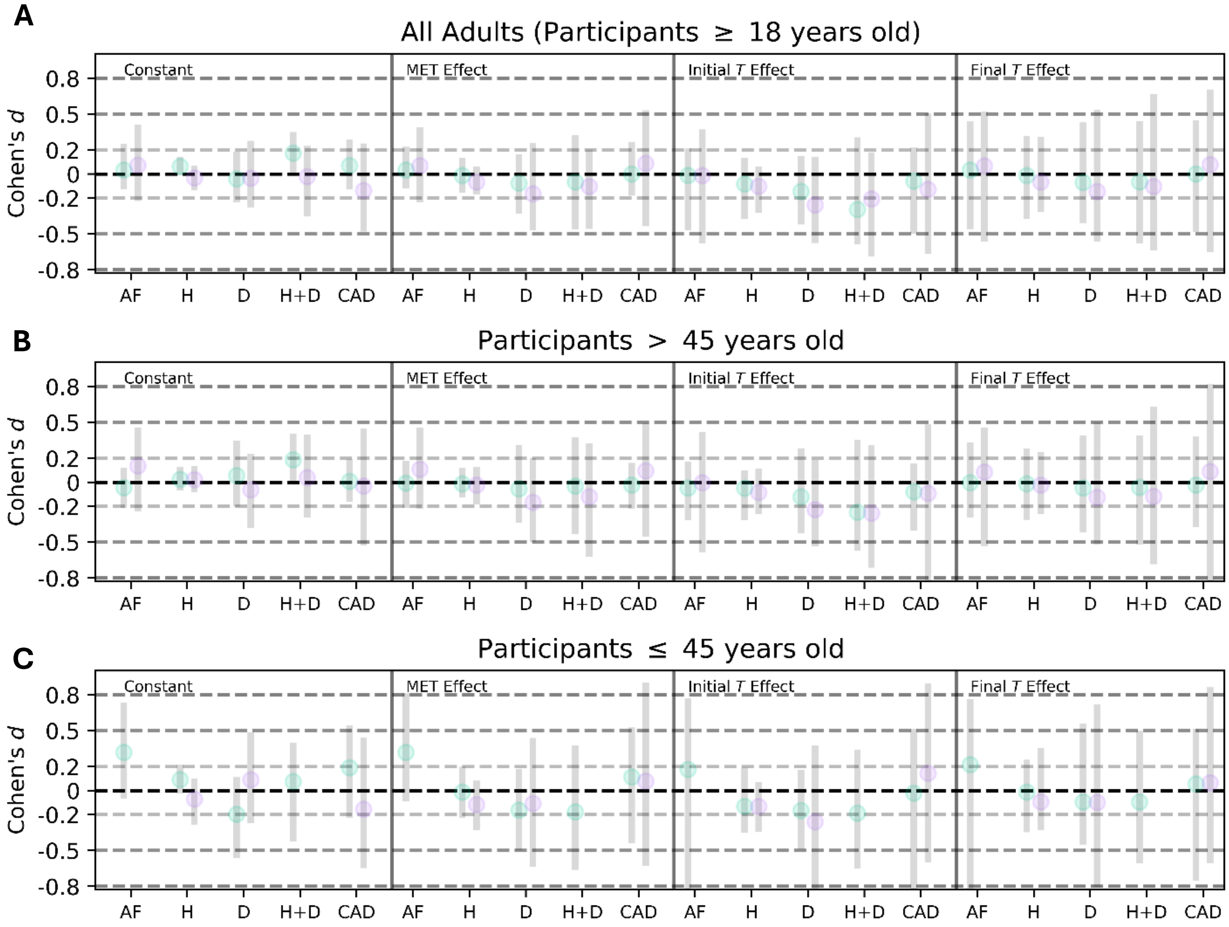

Supplementary Figure 3. **Cohort separability by multilinear model parameters across multiple cardiovascular diseases**. Median Cohen's *d* values (circular scatterplot markers) of male (cyan) and female (purple) model coefficients based on comparing a sex-matched cohort with no reported conditions against cohorts with reported Atrial Fibrillation (AF), Hypertension (H), Diabetes Mellitus (D), both H and D (H+D), and Coronary Artery Disease (CAD). The black lines through each median circular marker reflect the 2.5th percentile (lowest end) and 97.5th percentile (highest end) of the distribution of odds-ratios when comparing the cohort with a condition against a size-matched random selection from the control cohort (with replacement) 1000 times. Markers that are solid (not transparent) reflect that the original Mann-Whitney U test to compare cohorts was significant after Bonferroni correction *and* the 2.5th and 97.5th percentiles of the distribution of odds-ratios are either both *above* 0 (the parameters are greater in the control cohort) or are both *below* 0 (the parameters are lower in the control cohort). Each subplot is then an age-separated cohort of either the original multilinear model parameters (left subplots) or the time-dependent model (right subplots). **A.** Multilinear model parameters from participants who were at least 18 years old. **B.** Multilinear model parameters from participants who were at least 46 years old. **C.** Multilinear model parameters from participants who were at most 45 years old.
